# Supplementary material for: Salmonella-vectored vaccine delivering three Clostridium perfringens antigens protects poultry against necrotic enteritis
Source: PLoS One. 2019 Feb 12;14(2):e0197721. doi: 10.1371/journal.pone.0197721 (PMC6372158; doi:10.1371/journal.pone.0197721)
Supplement: S1 Fig — Strains were grown with aeration in LB supplemented with 0.1% arabinose and 0.1% mannose. A. Optical density measurements; B. Colony forming units obtained by plating onto LB + arabinose at the indicated times. (PPTX) [file pone.0197721.s001.pptx]

## Slide 1
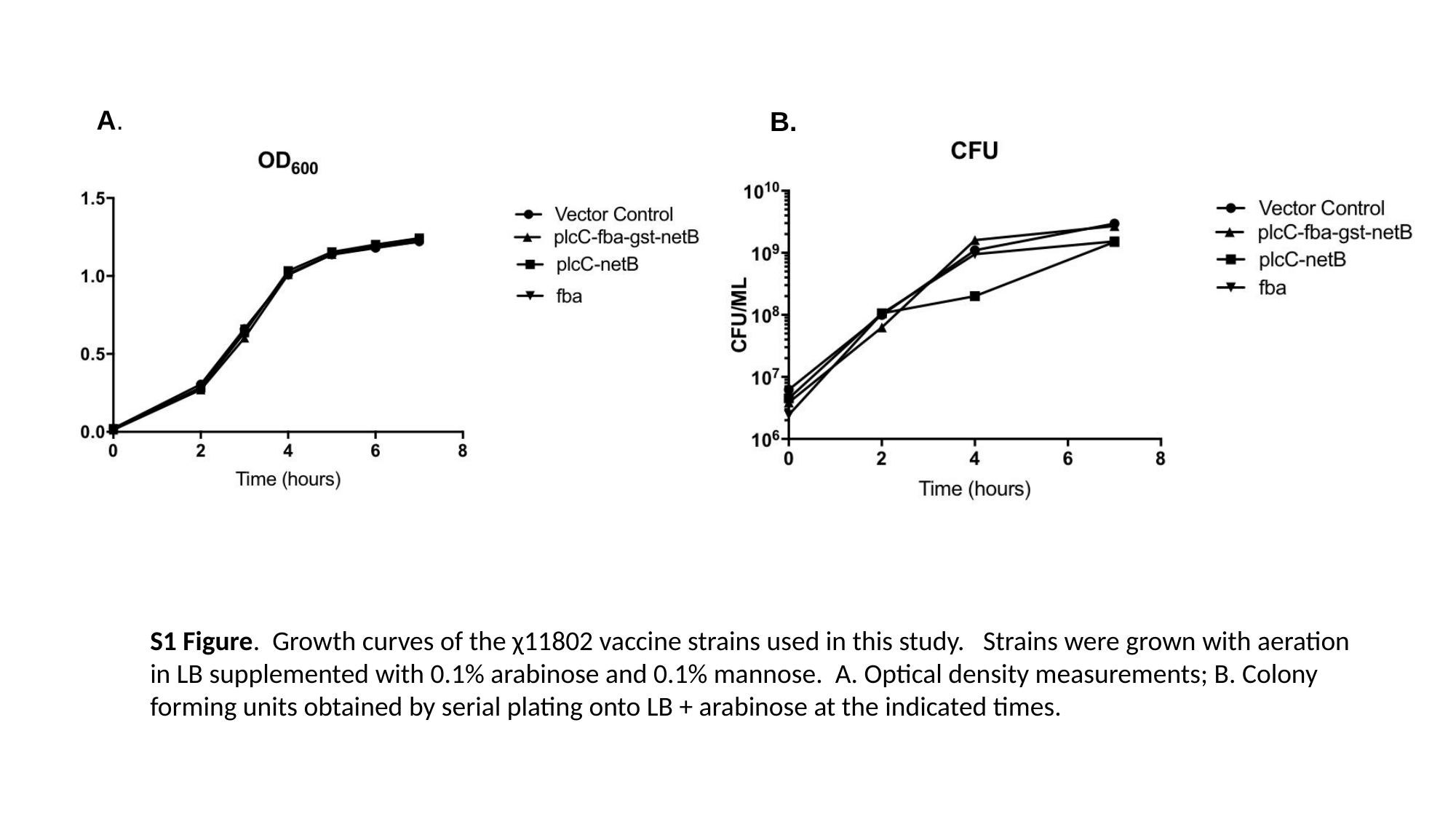

A.
B.
S1 Figure. Growth curves of the χ11802 vaccine strains used in this study. Strains were grown with aeration
in LB supplemented with 0.1% arabinose and 0.1% mannose. A. Optical density measurements; B. Colony
forming units obtained by serial plating onto LB + arabinose at the indicated times.
