# Supplementary material for: Salmonella-vectored vaccine delivering three Clostridium perfringens antigens protects poultry against necrotic enteritis
Source: PLoS One. 2019 Feb 12;14(2):e0197721. doi: 10.1371/journal.pone.0197721 (PMC6372158; doi:10.1371/journal.pone.0197721)
Supplement: S2 Fig — Cells were incubated with pre-immune sera (Pre-Bleed), secondary antibody only (2o α) or the indicated antisera with or without prior incubation with the indicated recombinant proteins as outlined in the Materials and Methods section. (PPTX) [file pone.0197721.s002.pptx]

## Slide 1
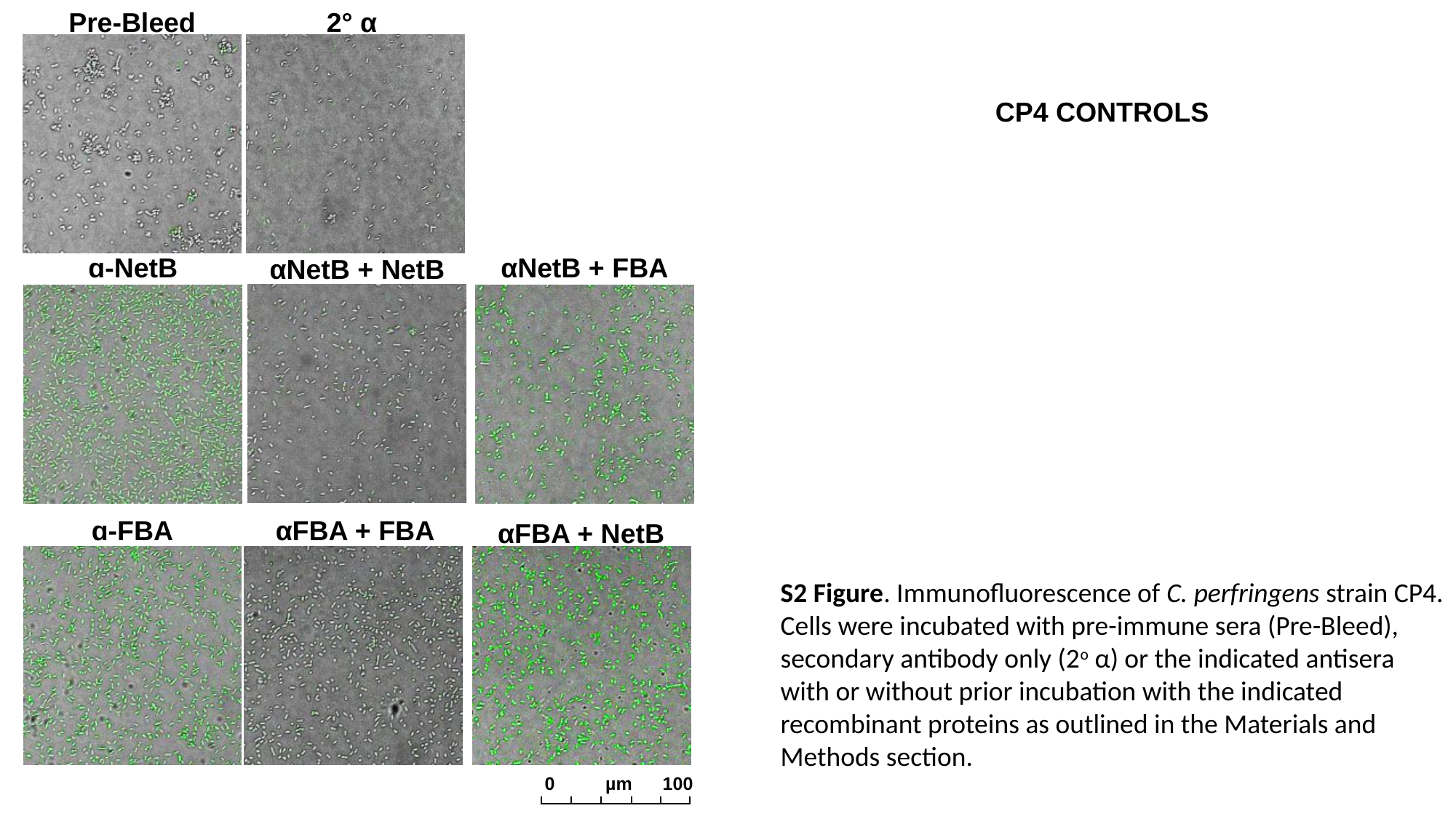

Pre-Bleed
2° α
ɑ-NetB
αNetB + FBA
αNetB + NetB
ɑ-FBA
αFBA + FBA
αFBA + NetB
CP4 CONTROLS
S2 Figure. Immunofluorescence of C. perfringens strain CP4.
Cells were incubated with pre-immune sera (Pre-Bleed),
secondary antibody only (2o α) or the indicated antisera
with or without prior incubation with the indicated
recombinant proteins as outlined in the Materials and
Methods section.
0 µm 100
